# Automatic Bounding Box Annotation with Small Training Datasets for Industrial Manufacturing

**DOI:** 10.3390/mi14020442

**Published:** 2023-02-13

**Authors:** Manuela Geiß, Raphael Wagner, Martin Baresch, Josef Steiner, Michael Zwick

**Affiliations:** 1 Software Competence Center Hagenberg GmbH, Softwarepark 32a, 4232 Hagenberg, Austria; 2 KEBA Group AG, Reindlstraße 51, 4040 Linz, Austria

**Keywords:** automatic object annotation, image annotation, object detection, AutoML, deep learning, Industry 5.0

## Abstract

In the past few years, object detection has attracted a lot of attention in the context of human–robot collaboration and Industry 5.0 due to enormous quality improvements in deep learning technologies. In many applications, object detection models have to be able to quickly adapt to a changing environment, i.e., to learn new objects. A crucial but challenging prerequisite for this is the automatic generation of new training data which currently still limits the broad application of object detection methods in industrial manufacturing. In this work, we discuss how to adapt state-of-the-art object detection methods for the task of automatic bounding box annotation in a use case where the background is homogeneous and the object’s label is provided by a human. We compare an adapted version of Faster R-CNN and the Scaled-YOLOv4-p5 architecture and show that both can be trained to distinguish unknown objects from a complex but homogeneous background using only a small amount of training data. In contrast to most other state-of-the-art methods for bounding box labeling, our proposed method neither requires human verification, a predefined set of classes, nor a very large manually annotated dataset. Our method outperforms the state-of-the-art, transformer-based object discovery method *LOST* on our simple fruits dataset by large margins.

## 1. Introduction

Reconfigurability and adaptability have become a major factor in small-lot industrial manufacturing in the era of smart factories and Industry 5.0 [1]. Especially in the context of human–robot collaboration (Cobots, [2]; Industry 5.0), a robot needs to be able to quickly adapt to the changing demands of a human operator. This is a typical challenge in the widespread field of bin-picking tasks [3,4]. In such a setting, the ability to detect objects in the working area is crucial for a robot to react to an operator’s commands. State-of-the-art (deep learning-based) object detection models typically operate under a closed-world assumption; that is, all object classes of interest are known beforehand and present in the training dataset. In practical applications, however, a robot often needs to react to a changing environment, i.e., it is confronted with an open-world setting (open-world/set recognition, [5,6,7]), where novel object classes need to be recognized that were not present in the dataset used for model training. Besides the ability for fast retraining of the object detection model [8], the question of how much data are needed for training is of great importance in a challenging environment such as industrial manufacturing [9]. Notably, automatic labeling of new data is essential for a robot to adapt to changing environments in order to avoid the usually time-consuming and therefore often expensive manual labeling process.

In recent years, significant efforts have been made to automate all aspects of the traditional machine learning pipeline (AutoML, [10]). In object detection, where manually labeling bounding boxes is especially time consuming, the potential benefits of an AutoML-based approach are particularly significant and a crucial factor in enabling human–robot collaboration. However, the existing approaches for automatic bounding box annotation are typically either trained on a fixed set of classes or rely on human verification while annotating datasets in an iterative manner, often requiring large manually annotated datasets as a prerequisite (see Section 2).

**Figure 1:**
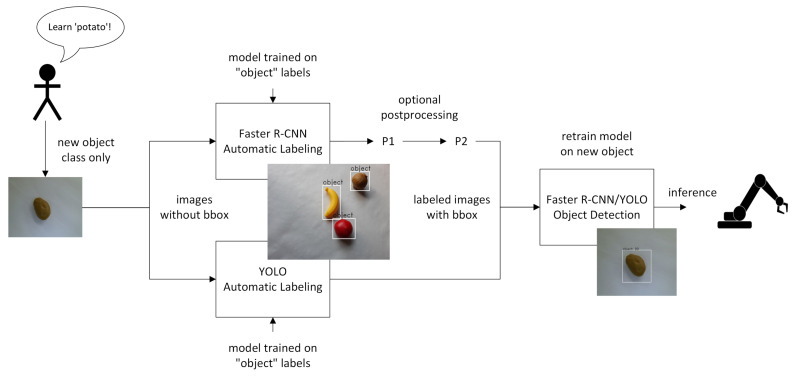
Overall workflow of object registration. A human operator initiates the training of a new object by only presenting the new object and providing the label without bounding boxes. Without any further human intervention, bounding boxes are then inferred by models trained on images using only an "object" label. Optionally, additional post-processing is applied which consists of the two steps (P1) and (P2) for merging of multiple bounding boxes and slightly increasing bounding boxes (see Section 3.2 for a detailed description). The training images with these automatically inferred bounding boxes are used to train the final object detection model.

In this work, we propose a deep learning-based automatic labeling approach which neither requires human verification, a predefined set of classes, nor a very large manually annotated dataset. Our approach is especially suitable for fast retraining of object detection models in human–robot collaboration settings for smart factories. More precisely, we focus on a use case with a homogeneous (but not necessarily simple) background (see Section 3.3.2 for an example), where new objects are incrementally learned with a human operator simply initiating the training of new classes while only providing the new class label. This workflow is visualized in Figure 1. We show that classical state-of-the-art object detection models, such as Faster R-CNN and YOLO, can be used to distinguish new objects from such a homogeneous background and that a small initial dataset is sufficient for training the model for this task. In particular, our proposed method does not require retraining the model for detecting previously unseen classes. Using object detection approaches in our setting has the added advantage that a single model architecture can be used for both tasks of bounding box annotation and object detection, which is beneficial, e.g., for use on edge devices. We show that our proposed method outperforms the unsupervised state-of-the-art object discovery method *LOST* [11] which, similar to our approach, is supposed to also detect unknown classes that have not been part of a previous training dataset.

Our work is structured as follows: Section 2 gives a short overview of deep learning-based object detection methods relevant to our approach, learning on small datasets and the current state-of-the-art in automated data annotation. Section 3 describes the use case and test setting that our approach was deployed on, the overall workflow, as well as the object detection architectures used during analysis. Section 4 gives an overview of the datasets used (one specifically generated for this work, one publicly available) and details the results of our experiments. Finally, Section 5 highlights our key findings and discusses potential further improvements to our work.

## 2. Related Work

### 2.1. Object Detection

Deep learning has been a driving force in the field of machine learning that has revolutionized many tasks such as object detection [12], i.e., the task of classifying and localizing objects in images. Object detection methods are utilized in various applications such as face recognition [13], self-driving cars [14], and fruit recognition in the context of robotic harvesting [15]. Although the history of multilayer networks dates back to the middle of the 20th century, deep learning has only recently become popular with the development of high performance parallel computing (e.g., GPU clusters) and the availability of large annotated datasets such as ImageNet [16] for training large network structures [17]. One important breakthrough that marked a milestone for the wide application of deep learning methods and laid the groundwork for modern object detection methods was the development of AlexNet [18], a deep convolutional neural network (CNN) that achieved outstanding results in the popular ImageNet Challenge (ILSVRC) [16] in 2012. During the last decade, many CNN-based methods for object detection have been developed, which can be mainly categorized into two classes: the two-step region proposal-based methods, such as Faster R-CNN [19], FPN [20], and Mask R-CNN [21,22], and the one-step anchor box-based approaches, such as the YOLO-family [23,24,25] and SSD [26] (see, e.g., [17] for a detailed review). These two classes differ in their accuracy–speed trade-off, with anchor box-based methods having a much smaller inference time, while region proposal-based methods achieving higher accuracy [27,28]. However, recent results suggest that newer versions of YOLO can attain accuracy levels comparable to region proposal-based networks [29,30].

### 2.2. Small Datasets

Current object detection methods fall within the category of supervised learning algorithms with complex network architectures that typically include millions of learnable parameters and therefore require large labeled datasets for training [12,17]. Few-shot learning [31] is the research field which addresses the problem of learning high quality models from small sample sizes. In general, approaches to few-shot object detection [32,33,34] use an established architecture with pre-trained weights based on a large dataset with labeled images, and then train an adapted architecture and/or loss function on novel object classes with far fewer samples.

State-of-the-art few-shot object detection approaches fall into one of four categories. (1) Data augmentation [35] tries to enhance a small dataset by adding additional images generated from the original images using suitable transformations. (2) Transfer learning [36,37,38,39] tries to exploit knowledge gained from training a data-rich source task to improve the performance of a target task with only a few images. (3) Distance metric learning [40,41,42,43,44,45] uses a distance metric to map images into a lower-dimensional embedding space where similar samples according to the metric lie closer together than less similar ones. In the lower-dimensional feature space, a smaller dataset can then be trained with less risk of overfitting and better generalization performance. Finally, (4) meta learning [46,47,48,49] takes a number of meta datasets, e.g., the images belonging to each class in a training dataset, and learns how to generalize from one subset of classes to another. This knowledge can then be used to generalize to a previously unseen class with only a few samples, as the meta learner already knows how to generalize to a new class.

To improve the detection performance of small-scale datasets, BackgroundNet [50] uses samples of background images without any objects as additional inputs to a YOLO-based architecture, thus enabling the network to not only learn object features, but also to distinguish between objects and background. In addition, the strategy for which grid cells are responsible for bounding box prediction is adapted to counter the difficulty of learning the center point of objects when only a few samples are present in the dataset. Ref. [51] presents a case study for the visual inspection of electrical utility assets with few images taken by maintenance workers during routine inspections. The proposed framework is based on RetinaNet [28] and adopts several strategies (progressive resizing [52], learning rate finder [53], and range optimizer [54]) to improve the training speed and accuracy when training on a small dataset.

### 2.3. Bounding Box Annotation

The most popular open, large-scale datasets for object detection are the ImageNet [16], Pascal VOC [55], and MS COCO [56] datasets, all containing thousands of annotated images per class. However, the annotation of images is a time-consuming and therefore costly task. This raises the need for solutions to automated annotation of images. While the problem of automatic image annotation for classification tasks has been treated for more than two decades now (see, e.g., [57] for a detailed review), the more challenging task of bounding box annotation has only come into focus in the last few years. The available methods for the latter task range from inferring the location of object proposals from edges [58], using predictions from a U-Net neural network as a basis [59], and training the detector model on a subset of manually labeled images [60]. Such methods have been used in various applications such as industrial visual inspection [61], radiology [59], 3D images [62,63], and object tracking in videos [64].

However, existing approaches typically rely on some human intervention to tackle the trade-off between accurate, manually labeled training data and the prediction accuracy of the trained models. In most cases, this intervention is some sort of verification [58,65] or manual correction [66] of the inferred bounding boxes. Such approaches typically rely on state-of-the-art object detectors such as Faster R-CNN or Fast R-CNN which are retrained many times to predict bounding box annotations that are then corrected by a human operator to serve as training data in the next training iteration cycle [58,60]. Similarly, there exists commercial products such as *Amazon SageMaker Data Labeling* (https://aws.amazon.com/sagemaker/data-labeling), *Microsoft’s Azure*, or *Superb Labeling* (https://www.superb-ai.com, accessed on 1 February 2023) that also offer AI-assisted bounding box annotation; however, they also heavily require human verification of the proposed predictions. These products usually require annotated training datasets (often more than 1000 images) as a prerequisite or are restricted to some predefined classes due to pre-training on some dataset COCO. Moreover, the underlying AI model architecture often remains unclear. Another strategy is active learning [67], which reduces the annotation costs by sampling the most informative unlabeled images that are then labeled by a human operator. This decreases the amount of manual labeling but is usually computationally expensive due to the sampling step. The related assistive learning workflow of [68] reduces the computational costs by including some contextual information in the sampling method; however, it still requires a human-in-the-loop. Furthermore, there exists labeling approaches using attention maps to extract bounding boxes; however, the accuracy is limited and a manual control step is required [59]. Another group of approaches address the problem of image co-localization; that is, objects are localized based on object similarity by using images containing objects of the same category [69,70,71]. This, however, limits the application to new objects not similar to available data. The self-supervised, transformer-based method *LOST* [11] is supposed to generalize well to localizing objects that have not been seen during training; however, as we will show in Section 4, this ability is limited.

For our use case of continuously learning new objects in a short time, manual correction steps are not possible due to time restrictions. Moreover, we cannot guarantee that new objects are similar to already known objects, as required by, e.g., co-localization approaches, or fall into a predefined set of classes. On the other hand, we have seen in previous experiments during our project that, for good training results, the bounding boxes do not have to be perfectly accurate in the sense that some pixels can be missed in some cases. Our approach of training object detector models to distinguish between objects and background does meet these requirements and performs well for detecting unknown objects. Our method has parallels to existing ones in the sense that it relies on state-of-the-art object detection architectures but, in contrast to, e.g., [58,60,68], it applies small changes in the training data and uses optional post-processing steps instead of including human correction steps.

## 3. Material and Methods

One of the main problems of annotating images for object detection tasks is the correct identification of the bounding box. In this work, we consider the use case of a *homogeneous* background, which allows us to use state-of-the art object detection methods to distinguish the object from the background.

### 3.1. Use Case

**Figure 2:**
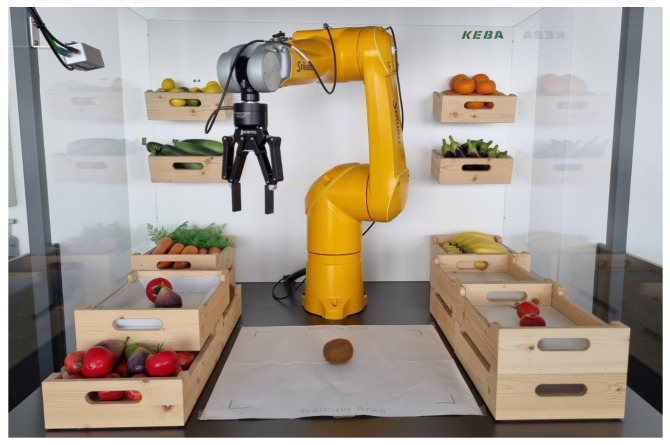
Showcase to demonstrate fast retraining of new object classes: A robot sorting different types of fruits. The left box holds a mixture of different fruits, from which the robot picks up specific ones and transfers them to the correct basket on the right. The new object needs to be placed in the center of the white area by a human operator for the generation of training images. These images are taken by a camera at the robot’s gripper while the robot is moving around the object. (Source: KEBA Group AG.)

As a use case we consider a robot that has to recognize objects and sort them into predefined boxes (see setup in Figure 2). In addition, our setup is that of an open world; that is, the robot has to learn new objects at later time points without forgetting the old ones. For this object detection task, we assume that a human operator initiates the training of the new object by presenting the object to a camera and providing the name of the object. The rest of the pipeline, that is the generation of training images, labeling of the images, and training of the model, is then performed automatically. In this work, we focus on the automatic annotation of the training and validation images. The overall workflow of our object registration pipeline is shown in Figure 1. For a more detailed description of the whole pipeline including automatic image generation and the training process, we refer to our recent contribution [8].

The automatic annotation of images for object detection is in general not an easy task. First, it is often not known at which time point a new object class has to be learned and what the label of the new object class is. The identification of an object to be unknown is a non-trivial task, which is not inherently captured by the design of current deep learning models [6]. Second, for retrieving the corresponding bounding box, the model has to distinguish a new object from some—often complex and previously unknown—background. Our use case differs from general object detection tasks in the following main points: (i) there is a human signal whenever a new object has to be learned, (ii) the name of the new object class is given to the AI model, (iii) the new class is trained on images contains only a single object, and (iv) the background remains approximately the same, i.e., we can speak of a *homogeneous* background. By *homogeneous* we do not necessarily mean that the background must be monochromatic or exactly the same in each image (e.g., a white background in all images) but we also speak of an homogeneous background if, for instance, each image contains different parts of a possibly complex environment (see, e.g., the iCubWorld dataset in Section 3.3). This use case of a homogeneous background represents the realistic scenario of a robot being used in a static environment, e.g., a robot installed at a fixed position in a factory.

### 3.2. Our Approach

The task of annotating an image for object detection consists of two separate subtasks: (1) determining the class label and (2) finding the bounding box. In our use case, the image that needs to be annotated contains exactly one object and the label of its class is provided by the human operator. Thus, the class label is already given and it remains to find the corresponding bounding box coordinates. For this task, our approach is to train some state-of-the-art object detection method for background separation and distinguish the new object from the background. As training data, we provide the network with annotated images of different objects in the same homogeneous background setup. In contrast to conventional training of object detection models, in our approach, the same label “object” is given to each object in the training and validation data. After successful training, the bounding box coordinates of the object in the test image can now be extracted by applying the trained object detection model on the image and retrieving the bounding box of the resulting prediction. For some models, we found it to be beneficial to include two additional post-processing steps to further refine the bounding box (for examples also see Section 4.1):

(P1) If the model erroneously predicts more than one bounding box per image, merge all bounding boxes into one, which is the smallest bounding box containing all others;
(P2) Add some additional slack; that is, increase the bounding box by a few pixels on each side.

This workflow will be applied to two different state-of-the-art object detection networks, the two-step Faster R-CNN model and one representative from the one-step YOLO family. These architectures have been chosen since they are the two most used deep learning-based object detection approaches differing with respect to their performance–speed trade-off, where Faster R-CNN is more accurate and robust while YOLO architectures are typically faster.

#### 3.2.1. Faster R-CNN

**Figure 3:**
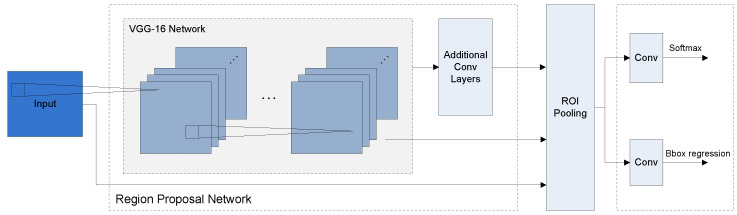
Architecture of the adapted Faster R-CNN model with a VGG-16 backbone. Figure reused from our recent work [8].

The architecture of the Faster R-CNN network consists of two stages: (i) a region-proposal network with a feature extractor network (in our case this is the VGG-16 backbone) followed by additional convolutional layers and (ii) the network’s head consisting of two outputs, one for object classification and the other for bounding box regression. These two parts of the network are connected by an ROI Pooling Layer. The second part of the network typically consists of fully connected layers. However, due to some technical limitations in the overall project of our use case (see below), we replaced these fully connected layers by convolutional layers; more precisely, our network’s head consists of one single convolutional layer with Softmax activation for the classification and one convolutional layer with linear activation for bounding box regression. These technical limitations result from running the inference of our trained Faster R-CNN network on an FPGA using the deep neural network development kit (DNNDK) (https://www.xilinx.com/support/documentation/user_guides/ug1327-dnndk-user-guide.pdf, accessed on 1 February 2023) from Xilinx (see also [8] for a description of our full pipeline). The architecture is shown in Figure 3.

#### 3.2.2. YOLOv4-p5

**Figure 4:**
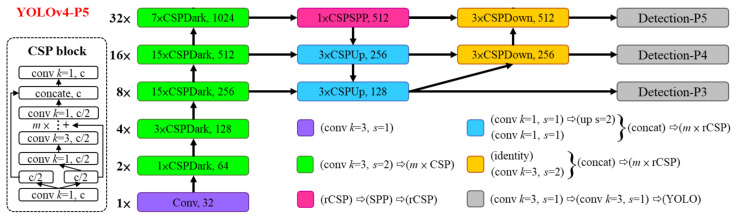
The architecture of YOLOv4-p5. Figure adapted from [25].

From the YOLO family of model architectures, we chose the recently developed Scaled-YOLOv4-p5 [25] which was among the best performing YOLO architectures at the time this study was performed. The framework is based on the large branch of the official pytorch implementation (https://github.com/WongKinYiu/ScaledYOLOv4, accessed on 1 February 2023). The architecture of the Scaled-YOLOv4-p5 model is summarized in Figure 4. It consists of 32 modules in total with 476 layers containing about 70 million parameters.

### 3.3. Datasets

The workflow presented in the previous section has been applied to two different datasets with a homogeneous background. They are described in more detail in the following sections and summarized in Table 1.

#### 3.3.1. Fruits

**Figure 5:**
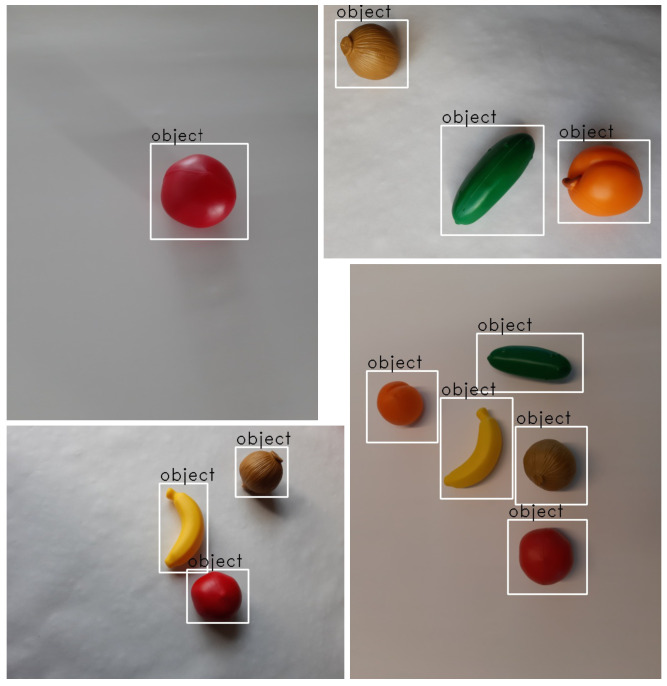
Annotation examples from our fruits dataset. Annotations have been generated manually using LabelImg. A piece of paper serves as background; however, the lighting differs and often induces shadows. Figure reused from our recent work [8].

**Figure 6:**
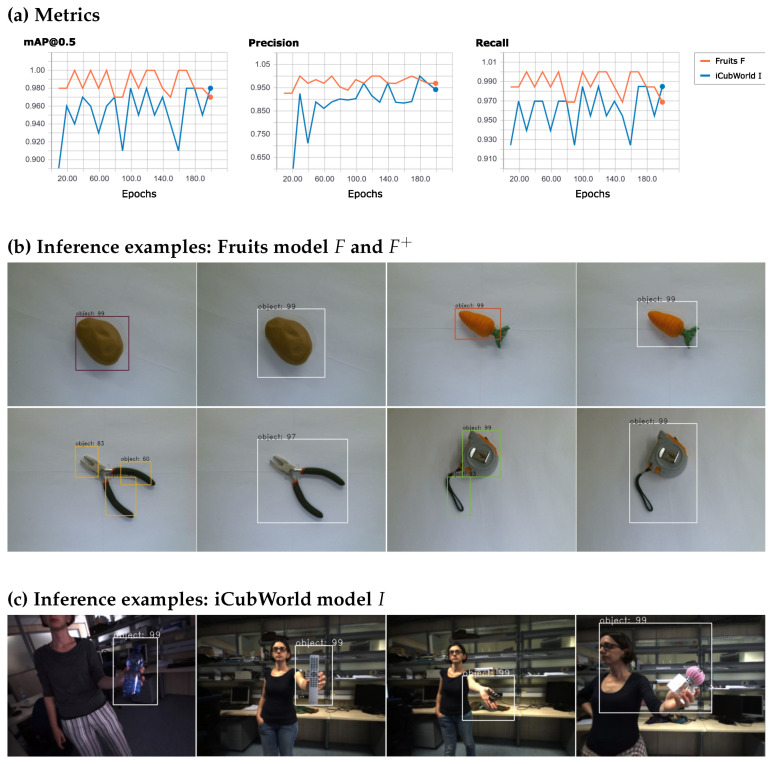
Results on the fruits dataset and the iCubWorld dataset using Faster R-CNN. For both, the corresponding models *F* and *I* has been trained for 200 epochs. Panel (**a**): The metrics (mAP@0.5, recall and precision) on the validation datasets are calculated after each 10th epoch (orange line: fruits, blue line: iCubWorld). For both models *F* and *I*, the metrics reach high values after only a few epochs. Panel (**b**): Inference examples of *F* and $F^{+}$ on the fruits test dataset. For each object, the results of *F* and $F^{+}$ are shown on the left and right, respectively. The potato is well annotated by both models, while the carrot benefits from the additional slack in $F^{+}$. For the pliers and the tape, *F* detects multiple bounding boxes resulting in a very good annotation of $F^{+}$. Figure reused from our recent work [8]. Panel (**c**): Inference examples of $I^{+}$ on the iCubWorld test dataset. The first two examples are well annotated, while the inferred bounding boxes in the remaining examples are too large due to inclusion of the human hand (hair clip) and incorrectly detected boxes merged with correct boxes (perfume). More detailed results on the absolute numbers of correct and incorrect annotations can be found in Figure A1.

This dataset has been created during the course of one of our industrial projects. It has been inspired by a typical bin-picking application in the industrial domain: bin-picking of fruits and vegetables [72]. The dataset consists of 330 images of five different imitation fruits and vegetables made of plastic (apricot, banana, cucumber, onion, and tomato) on a white background. Approximately half of these images contain only one object, the rest contain a mix of different object classes with up to four objects. The pictures are of size 708 × 531 and 576 × 432 resp. 531 × 708 and 432 × 576, and have been manually annotated using the labeling tool LabelImg (https://github.com/tzutalin/labelImg, accessed on 1 February 2023) (see Figure 5 for some examples). These data are split into training and validation datasets (approximately 90% and 10% of the images, respectively). As a test dataset we used 100 images of size 800 × 600, each containing exactly one object on a white background. This dataset contains 20 different classes (five images each), including the training objects and new objects such as other fruits and vegetables, pliers, measuring tape, etc. (see Figure 6b).

#### 3.3.2. iCubWorld

As a second dataset, we chose the iCubWorld (https://robotology.github.io/iCubWorld/, accessed on 1 February 2023) dataset [73]. This choice was made mainly because iCubWorld is one of the few publicly available datasets fitting our definition of homogeneous background which, in addition, is not monochromatic and therefore serves as a good test setup for analyzing to what extent our approach works on more complex backgrounds. Besides, the iCubWorld dataset has been generated with the same rationale of automatic data creation on which our study is based. Although the background in these images does not represent an industrial scenario, there are still similarities (shelves and color/lightning) that might also appear in certain industrial environments.

**Figure 7:**
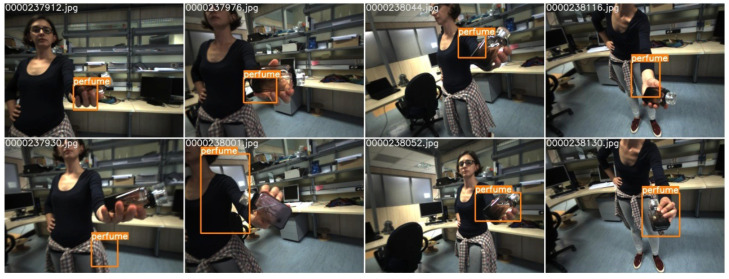
Example images from the iCubWorld dataset including the ground truth annotations that have been automatically generated from a human–robot interaction. In many cases the object has not or has only inaccurately been localized.

The iCubWorld dataset contains more than 400k images of 20 different classes. In contrast to the fruit dataset, the background in the iCubWorld dataset is not exactly the same in all images, but homogeneous in the sense that all pictures have been taken in the same environment, i.e., at different positions in one university lab and with the object held by the same person with changing clothing. The data contain annotation information for object detection, where the annotations also contain information about different poses such as “Mix”, “2D rotation”, etc., describing how the images change from one frame to the next. In the original dataset, these annotations have been automatically generated by a robot during a human–robot interaction, where the human provided the label verbally and showed the object in their hand. The robot then localizes the object by tracking either motion or depth information. Example images, including the ground truth annotations, are shown in Figure 7. It can be seen that the annotations are not optimal in many cases. For this reason, we again used LabelImg for manually creating our own ground truth annotations. We randomly chose 659 images of seven object classes (mug, pencil case, ring binder, soap dispenser, soda bottle, squeezer, and sunglasses) with 83–105 images per class and split this into training and validation datasets (90% and 10% of the images, respectively). As a test dataset we used a different subset of 82 images that contained all 20 object classes.

## 4. Results and Discussion

### 4.1. Experiments with Faster R-CNN

For the experiments with our adapted Faster R-CNN architecture (see Figure 3), we used the pretrained weights from the Keras Applications Module (https://github.com/fchollet/deep-learning-models/releases, accessed on 1 February 2023) for the VGG-16 backbone. We then trained two models: the model *F* on the fruits dataset and the model *I* on the iCubWorld dataset (see Table 1). Both models were trained for 200 epochs without freezing layers. We also tried freezing the VGG-16 backbone; however, the results were not satisfying, therefore we do not further discuss this here.

**Figure A1:**
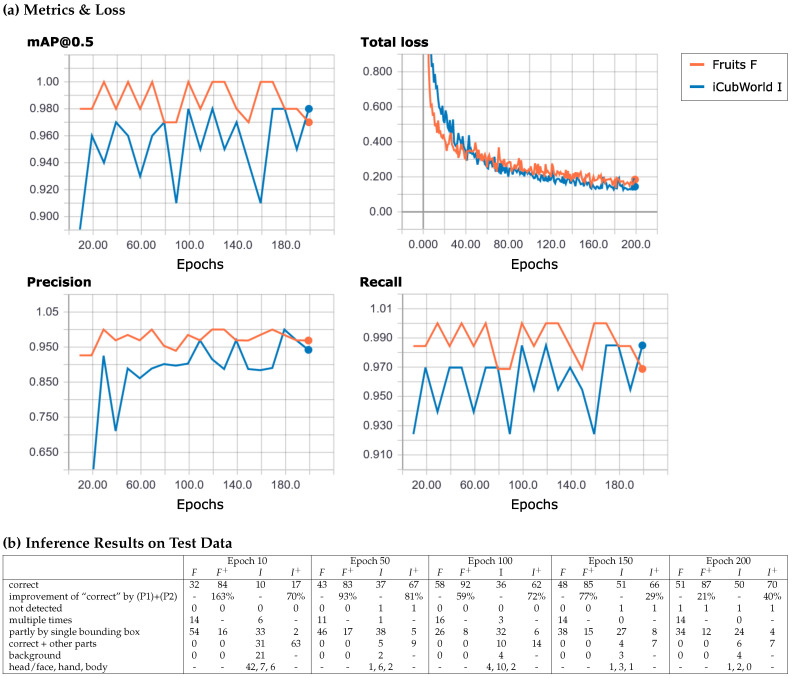
Results on the fruits dataset and the iCubWorld dataset using Faster R-CNN. For both datasets, the Faster R-CNN architecture was trained for 200 epochs. Panel (**a**): The metrics (mAP@0.5, recall, and precision) of the validation datasets are calculated after each 10th epoch (orange: fruits and blue: iCubWorld). For both datasets, the metrics reach high values after only a few epochs. The loss of the training dataset is shown after each epoch. Panel (**b**): Inference results on the test datasets of 100 (fruits) and 82 (iCubWorld) images, respectively, both containing more object classes than the training data. For more details about the different categories see the text.

The metrics (mAP@0.5, recall, and precision) were calculated after each 10th epoch. Here, the mAP@0.5 refers to the mean average precision at IoU threshold 0.5 (Pascal VOC Challenge) and recall and precision are calculated as $\frac{TP}{TP+FN}$ and $\frac{TP}{TP+FP}$ (TP = true positives, FP = false positives, and FN = false negatives), respectively. As can be seen in Figure 6a, all metrics reach very high values after only a few epochs. Model *F* performs slightly better than model *I*, where *F* reaches mAP@0.5 values between 0.97 and 1.0 during the entire training period, starting with 0.98 at epoch 10. The lower performance of model *I* is not surprising due to the more complex background in the images of the iCubWorld dataset. However, *I* also shows good performance with an mAP@0.5 constantly higher than 0.91 after only 20 epochs. Note that recall and mAP show similar variation for both models. This is due to the fact that the average precision (AP), i.e., the area under the precision–recall curve, and the recall are almost identical in our case since the precision is very close to 1. Inference examples of *F* on test data (see Figure 6b) show that (i) the predicted bounding box is often tight around the object with sometimes missing parts of the object (see, e.g., carrot) and (ii) in some cases the object is not detected by one single bounding box but several boxes cover different parts of the object. These multiple bounding boxes particularly occur for objects whose form is very dissimilar to the forms of the objects in the training dataset (e.g., pliers and tape with long, almost separated parts). In order to reduce such errors, we applied the two post-processing steps (P1) and (P2) introduced in Section 3.2 to merge multiple bounding boxes and enlarge the boxes by some additional slack (15 and 10 pixels for the fruits and iCubWorld datasets, respectively). The resulting models are denoted by $F^{+}$ and $I^{+}$ for the fruits and the iCubWorld datasets, respectively. Although the performance of model *F* is high, even after only a short amount of training time, these post-processing steps further increase the number of good annotations from 32 to 84 out of 100 test images at epoch 10 and from 51 to 87 at epoch 200 (see Figure A1 in the Appendix A for a detailed list of the absolute numbers). The remaining 12 cases in epoch 200 of $F^{+}$, in which the object is only partly covered by the inferred bounding box, are mostly examples similar to the carrot shown in Figure 6b, where the bounding box misses only a tiny fraction of the object. However, for many applications, such as our use case of incremental learning of new objects, this small lack of impreciseness does not pose a problem, since we observed that usually each part of an object is detected in a decent amount of training images such that, in total, all parts are seen by the model during training. On the other hand, we found in subsequent experiments that a slightly enlarged bounding box, as it is for instance the case for the potato in $F^{+}$ shown in Figure 6b, does not result in poor training results for the new object as long as tight bounding boxes are not an urgent requirement in the particular application. Note that the model shows slightly better performance at epoch 180 than 200. However, as this difference is very small, we still chose to evaluate after 200 epochs here to be in accordance with the more detailed results in Figure A1, where an evaluation with even smaller epoch intervals also covering epoch 180 would have been infeasible.

**Figure 8:**
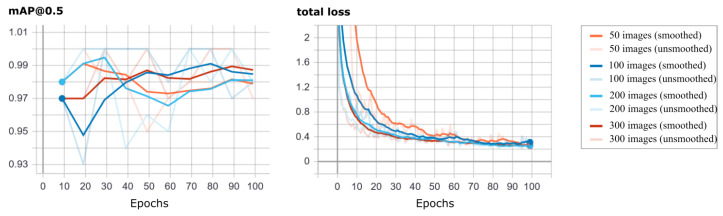
Comparison of different training dataset sizes. The Faster R-CNN model $F^{+}$ is trained for 100 epochs on subsets of the fruits dataset with 50, 100, 200, and 300 images. Shown are the mAP@0.5 (left column) and the value of the Faster R-CNN loss function (the total Faster R-CNN loss is a linear combination of four parts: the classification loss and the bounding box regression loss from both, the region proposal part and the Fast R-CNN layers) (right column) over time. As in Figure 6a, recall and precision are very similar to mAP@50; therefore, we did not include them here. The validation dataset is the same for all training runs. The shown graphics are exported from Tensorboard with a smoothing factor of 0.8 (dark lines), the unsmoothed values are included as light lines.

Moreover, we compared the effect of different sizes of the training dataset, namely 50, 100, 200, and 300 training images, on the performance of the $F^{+}$ model (see Figure 8). In our experiments, there is a trend of larger datasets leading to faster convergence to smaller loss values; however, the mAP@0.5 on the validation dataset is similar for all dataset sizes. Looking at the inference on the test dataset, which—in contrast to the validation dataset—also contains a large fraction of unknown classes, we observe that smaller datasets show slightly more problems with the detection of unknown classes, especially with the accuracy of bounding boxes, while missing detections are not significantly more frequent. For a dataset of 50 images, we find that ∼50% more bounding boxes miss (small) parts of the object compared to a dataset of size 300. Again, often the missing parts of the object are small, hence we do expect only small decreases in accuracy of follow-up object detection tasks that rely on these data annotations. We leave a deeper analysis of this aspect for future work.

The results for $I^{+}$ on test images of the iCubWorld dataset are shown in Figure 6c. Again, we found that the majority of objects are well detected in $I^{+}$ and the shift from *I* to $I^{+}$ largely improves the performance (see Figure A1 in Appendix A). We find that most objects are well detected even though they do not stand out clearly from the background (e.g., the soda bottle in Figure 6c) or were not in the training dataset (e.g., the remote in Figure 6c). A minority of the inferred bounding boxes either cover the object only partly (again, as in the case of $F^{+}$, only a few pixels are missing), are too large due to inclusion of the human hand (e.g., hair clip), or merge with incorrectly detected boxes (e.g., perfume). Moreover, for both models *F* and *I*, and thus also $F^{+}$ and $I^{+}$, it occurs only very rarely that an object is not detected at all.

**Figure 9:**
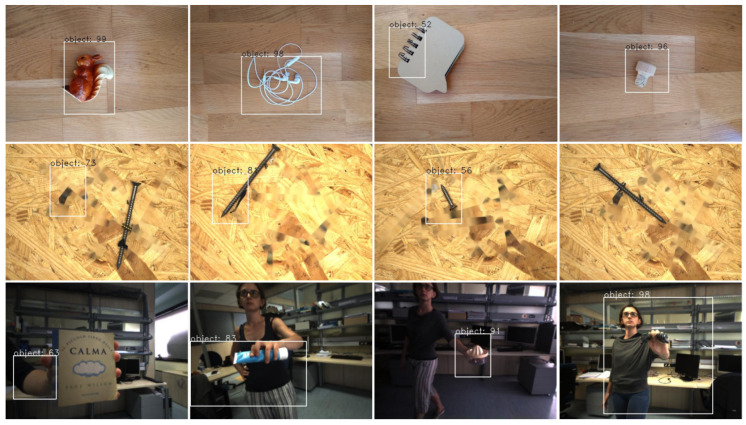
Inference results of the Faster R-CNN annotation model *F*${}^{+}$ trained on the fruits dataset for 200 epochs and applied to different datasets. The images in the first row are taken on a wooden floor, the second row contains images that have been modified from the original MVTec Screws dataset [74], and the last row shows examples from the iCubWorld data test. Figure adapted from our recent work [8].

Furthermore, we tested the transferability of such annotation models to a different background. For this, we applied the model $F^{+}$, that has been trained on fruits on a white background, to images containing various objects on different backgrounds. The results are shown in Figure 9 for images on a wooden floor (taken by us), modified images from the MVTec Screws dataset [74], and images from the iCubWorld dataset. It can be seen that the model transfers only poorly to other datasets. Applying $I^{+}$ to other datasets gives similar results on the MVTec dataset; however, the model performs well on the wooden floor dataset in five out of six images and mainly well on the fruits dataset as well, where the object is mostly detected but sometimes with an inaccurate bounding box. Repeating these experiments with *F* and *I* results in many inaccurate bounding boxes as well as detection of multiple boxes. In summary, these observations, together with the fairly good results of $I^{+}$ on the iCubWorld dataset, suggest that the model explicitly *learns* a given specific background but it can often be transferred to less complex backgrounds in combination with (P1) and (P2).

Finally, we compared our approach to the self-supervised transformer-based method *LOST* [11] requiring no labeled data, which outperforms other state-of-the-art methods in object discovery. This method is a good reference baseline for our experiments since it does not rely on human correction. Moreover, similar to our method, it can be applied to any image/object class without retraining. Apart from LOST, most other methods are unsuitable for direct comparison with our approach because they either require some human verification step or are trained on a set of fixed classes. Commercial, closed-source methods are not included into our comparison either. Besides the obvious costs, it is often not clear which are the underlying algorithms and, in addition, they often rely on large manually annotated datasets as a prerequisite. We applied LOST to the 100 images of the fruits test dataset. As shown in Table 2, our proposed method outperforms LOST by large margins (87% vs. 65% correct bounding box predictions). The number of undetected objects (12% vs. 1%) is significantly higher for LOST. In particular, while the 12% of partly detected objects for $F^{+}$ represent bounding boxes that are mostly only missing a few pixels, the 23% partly detected objects for LOST represent low quality detections with, e.g., bounding boxes covering one-third of the image, including large parts of the background. This suggests that, despite the simplicity of our use case, LOST fails to adapt to it without further retraining. Furthermore, the inference time per image is much smaller for $F^{+}$ than for LOST (∼1.5 s on a CPU vs. ∼3.5 s on a GPU, see Table 2 for more details). We would like to emphasize in this context that there exists much more efficient Faster R-CNN implementations than the one that we used as a basis for our code, hence the difference in inference speed between the two methods could be even larger. The training time could not be compared since LOST was designed and trained by its authors to be directly applicable to new datasets without retraining.

To conclude, we found that Faster R-CNN can be trained to distinguish (unknown) objects from a highly complex but homogeneous and specific background using a relatively small amount of training data and without human verification. The ability to transfer a trained model to a different dataset is limited but works in some cases where the background is less complex than in the training data.

### 4.2. Experiments with Scaled-YOLOv4-p5

**Figure 10:**
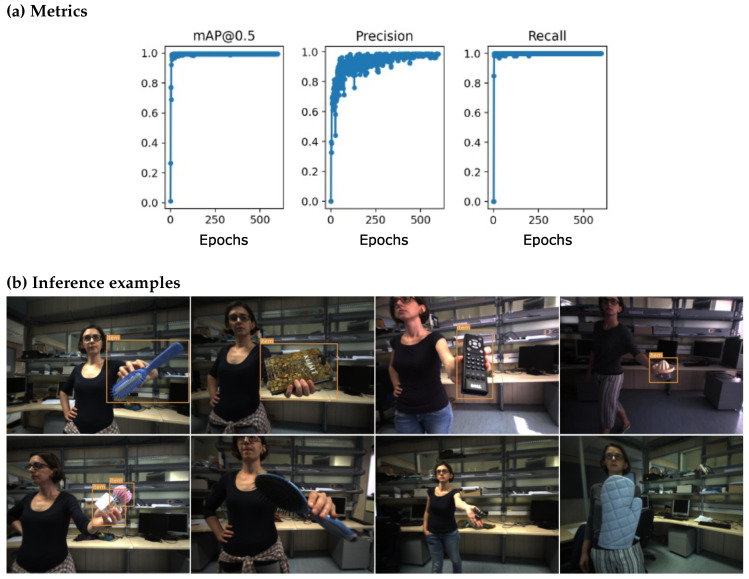
Results on the iCubWorld dataset using the Scaled-YOLOv4-p5 model. The model was trained for 200 epochs. Panel (**a**): The metrics (mAP@0.5, recall, and precision) on the validation datasets are calculated after each epoch and reach high values after only a few epochs. For more details see Figure A2. Panel (**b**): Inference examples on the iCubWorld test dataset at epoch 175. Most objects are detected well, some are not detected at all (5 out of 82). In contrast to Faster R-CNN, there are only few cases where multiple bounding boxes have been detected (1 out of 82) and bounding boxes are more accurate with only rarely missing parts of the object (8 out of 82), even though (P1) and (P2) are not applied.

Using a Scaled-YOLOv4-p5 model architecture pretrained on COCO (https://github.com/WongKinYiu/ScaledYOLOv4, accessed on 1 February 2023), we trained the model on this iCubWorld dataset. The results are shown in Figure 10.

As for the YOLO model, the metrics reach high values close to 1.0 after only a few epochs of training. In most cases, the model predicts good bounding boxes (see Figure 10b, top row, for some examples). Poor results appear in cases where no object is detected or one object is detected as two objects. Overall, in our test set with 82 images, only five objects were not detected and only once was an object detected as two objects. In contrast to the Faster R-CNN model, the bounding boxes are mostly very accurate although tight around the object (parts of the object were missed in only 8 out of 82 test images). The additional post-processing steps (P1) and (P2) were therefore omitted.

Finally, we tested the transferability of the model by applying it to datasets with different backgrounds. While the results of the MVTec dataset are similarly poor as in the case of the Faster R-CNN model, the predictions on the fruits dataset differ in comparison to $I^{+}$ (cf. Section 4.1) in the sense that the bounding boxes are similarly accurate yet tighter for the YOLO model but, as already seen for the iCubWorld test dataset, the YOLO model fails in detecting the object more often than Faster R-CNN. For the dataset with the wooden floor, the YOLO model gives comparable good results to $I^{+}$ if (P1) is applied, otherwise only three out of six images are predicted correctly, while in two cases the object is detected more than once.

In summary, the Scaled-YOLOv4-p5 architecture performs similarly well as the Faster R-CNN model in distinguishing (new) objects from homogeneous backgrounds. While the YOLO model fails to detect the object more often than the Faster R-CNN model, its predicted bounding boxes are tighter and often more accurate. Moreover, the YOLO model does not require the application of the post-processing steps (P1) and (P2) in most cases.

## 5. Conclusions

In this work, we discussed how to adapt state-of-the-art object detection methods for the task of automatic bounding box annotation in a use case where the background is homogeneous and a human collaborator only interferes by providing the object’s label. In contrast to other existing methods, our proposed approach does not require a human-in-the-loop for correcting predictions, thereby making the whole data annotation process much faster. Moreover, after being trained once on a relatively small manually annotated dataset for a given background, the model does generalize well to new classes without the need of retraining for those classes that have not been seen during training. In this context, we showed that our approach outperforms the state-of-the-art object localization method LOST.

In our experiments, we showed that both an adapted version of Faster R-CNN and the Scaled-YOLOv4-p5 architecture can be trained to distinguish unknown objects from a complex but homogeneous background using only a small amount of training data. In contrast to YOLO, the Faster R-CNN strongly benefits from using two post-processing steps that merge multiple bounding boxes and enlarge the final box. On the other hand, YOLO fails more often in detecting objects than Faster R-CNN. Our results suggest that both models explicitly learn the specific background in the training data, which limits the ability to transfer a trained model to a dataset with a different background. However, it seems that models trained on more complex backgrounds can be transferred to data with less complex backgrounds. A further analysis of the specific limits of this transferability and to what extent they can be defined would be an interesting question for future work. Moreover, it remains to further evaluate the minimum amount of required training data depending on the specific background as well as on transferability on different backgrounds.

An important advantage of using object detection methods for data labeling instead of standard instance or semantic segmentation approaches is the usually smaller inference time, which is crucial for fast generation of training data. Moreover, in our pipeline, it has the added advantage that a single model architecture can be used for both tasks of bounding box annotation and object detection. Nevertheless, it would be interesting for the future to also extend our approach to segmentation methods such as Mask R-CNN and possibly also develop corresponding post-processing steps such as (P1) and (P2) for Faster R-CNN.

## Figures and Tables

**Table 1:** Summary of (a) datasets and (b) models used in our study. The manually annotated images are split into 90% training and 10% validation data. In addition, a set of unlabeled images is used for testing. The Faster R-CNN models trained on the fruit and iCubWorld datasets are denoted by *F* and *I*, respectively. The models $F^{+}$ and $I^{+}$ denote the corresponding models after application of the post-processing steps (P1) and (P2).

(a) Datasets Used in Our Study					
**Dataset**	**No. Images**		**No. Classes**		**Training Classes Contained**
	**Train and val**	**Test**	**Train and val**	**Test**	**in Test Data**
Fruits	330	100	5	20	*Some* contained
iCubWorld	659	82	7	20	*All* contained
**(b) Faster R-CNN Models Used in Our Study**					
**Model**		**Training Dataset**		**Post-Processing (P1) and (P2)**	
*F*		Fruits		no	
$F^{+}$		Fruits		yes	
*I*		iCubWorld		no	
$I^{+}$		iCubWorld		yes	

**Table 2:** Comparison with LOST [11]. The inference results of the *F*${}^{+}$ model (at epoch 200) on the fruits test dataset are shown in comparison to application of the unsupervised object discovery method LOST on the same dataset. The Faster R-CNN-based model *F*${}^{+}$ achieves better results than LOST (87% vs. 65% correct bounding box predictions). The inference time is much smaller for $F^{+}$ on a CPU (Intel(R) Iris(R) XE Graphics) than for the LOST model on a GPU (NVIDIA GeForce MX450).

	*F* ${}^{+}$	LOST [11]
Correct prediction	87%	65%
Not detected	1%	12%
Partly detected	12%	23%
Inference time per image	∼1.5 s (CPU)	∼3.5 s (GPU)

**Figure A2:**
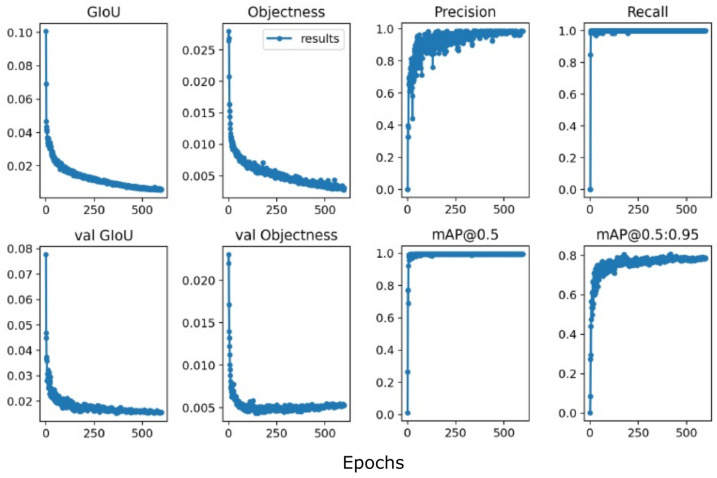
Training losses and metrics for the Scaled-YOLOv4-p5 trained on the iCubWorld dataset (see Section 3.3) for 200 epochs.

## Data Availability

The whole iCubWorld dataset is available here: https://robotology.github.io/iCubWorld/. The fruits dataset as well as the subset of the iCubWorld dataset used in this study (together with the created annotations) is available on our Github account: https://github.com/software-competence-center-hagenberg/Fruits-Dataset, accessed on 1 February 2023.

## Appendix A

In this section, we present more detailed results of the experiments in Section 4.

Figure A1 shows additional results for the experiments with the Faster R-CNN architecture. In addition to the metrics that have already been shown in Figure 6, it also contains the Faster R-CNN training loss. Moreover, detailed inference results for the models *F* and $F^{+}$ on the fruits test dataset and for *I* and $I^{+}$ on the iCubWorld test dataset are shown for different epochs from 10 to 200. The results are assigned to seven different classes:

Correct: Good detection, i.e., correct label, good bounding box, and no false positives/negatives.
Improvement in “correct” by (P1) and (P2): The improvement in the correctly identified bounding boxes from *F* to $F^{+}$ and *I* to $I^{+}$, respectively.
Not detected: The object is not detected.
Multiple times: Multiple bounding boxes covering the object or parts of it are inferred.
Partly by single bounding box: The object is detected by a single bounding box but the box misses parts of the object. We explicitly emphasize that this mostly corresponds to only very small missing parts (see also Section 4.1).
Correct and other parts: The object is well detected but other parts of the image are detected as well (see also the remaining two categories). We also count here objects that are detected by a bounding box that is at least twice as large as necessary, containing also, e.g., the person’s hand in the iCubWorld dataset.
Background: The background is detected. For the iCubWorld dataset, we do not include here the detection of (parts of) the person presenting the object. This is covered by the next category.
Head/face, hand, and body: The head/face, hand, or other parts of the body of the person presenting the object are detected (only applies to the iCubWorld dataset).

For the experiment with the Scaled-YOLOv4-p5, the complete set of metrics and losses is shown in Figure A2.
